# Fatigue and Sleep in Multiple Sclerosis Patients: A Comparison of Self-Report and Performance-Based Measures

**DOI:** 10.3389/fneur.2017.00703

**Published:** 2018-01-04

**Authors:** Madlen Paucke, Simone Kern, Tjalf Ziemssen

**Affiliations:** ^1^Centre of Clinical Neuroscience, Department of Neurology, University Hospital Carl Gustav Carus at the Technische Universität Dresden, Dresden, Germany

**Keywords:** multiple sclerosis-related fatigue, sleep, tonic/intrinsic/phasic alertness, pupillographic sleepiness test, multiple sclerosis

## Abstract

**Background:**

Multiple sclerosis (MS) patients suffer very often from MS fatigue and sleep problems. Despite the detrimental impact on the activities of daily living, a short and objective quantification of fatigue and sleep problems is currently lacking.

**Objective:**

The objective of the study was to systematically investigate tonic, intrinsic, and phasic alertness and the relationship of these performance-based measures with self-report measures of fatigue and quality of sleep.

**Methods:**

Thirty-three MS patients without (MS−) and 26 with selected comorbid disorders (MS+) and 43 healthy controls (HCs) performed the pupillographic sleepiness test (measuring tonic alertness) and the alertness subtest of the Test of Attentional Performance (measuring intrinsic and phasic alertness).

**Results:**

Self-reported and performance-based measures revealed poorer performance for both MS groups compared to HC. MS+ patients presented higher rates of MS fatigue, sleep problems and depressive symptoms but similar alertness scores compared to MS− patients. However, tonic alertness was only higher in MS− patients compared to HC. Intrinsic and phasic alertness correlated moderately with fatigue ratings.

**Conclusion:**

In the diagnostic process of MS fatigue and quality of sleep comorbid disorders (depression, anemia, thyroid dysfunction) and performance-based measures such as alertness should be considered in daily clinical practice.

## Introduction

Multiple sclerosis (MS) is an inflammatory disease of the central nervous system with a widespread demyelination and axonal loss (1). In addition to other neurological deficits, MS-related fatigue occurs in up to 90% of the patient population (2–4).

Multiple sclerosis-related fatigue is a heterogeneous clinical feature [see Ref. (5, 6) for an detailed multidimensional description] and is simply defined as a perceived subjective lack of (mental or physical) energy by the individual (6). New approaches describe MS-related fatigue as a complex symptom that includes three clinical different entities: asthenia (fatigue at rest), fatigability (fatigue with exercise), and worsening of symptoms with effort (5). Despite these entities, the pathophysiology underlying MS-related fatigue is still under investigation: Proinflammatory cytokines, overactivity of neural circuits, HPA axis involvement, and axonal injury are discussed [for an overview see Ref. (7)].

Multiple sclerosis-related fatigue negatively affects employment status as well as quality of life (8–11), and there is a strong need for a valid and reliable assessment (12). For the assessment of subjective perceived Fatigue, self-report measures are often used, because they are cheap and easy to administer. However, these measurements are vulnerable to a series of problems such as self-perception, social desirability, malingering, motives, memory processes, and overestimation (e.g., because of depression) or underestimation (e.g., because of anosognosia). Furthermore, a clear differentiation between primary (MS as cause for fatigue) and secondary fatigue (other conditions causing fatigue, such as depression, pain, e.g.) with these measures is not possible. That’s why the performance-based approach reflects an alternative way to objectively quantify MS-related fatigue (13). There have been some attempts to quantify the worsening of symptoms with effort by using sustained or repetitive muscle contractions or cognitive tests (14, 15). By using kinematic gait analysis (16) or hand dynamometer (14) correlations with physical but not with the cognitive dimension of subjective fatigue scales were found. In contrast, attentional functions and information processing speed, cognitive key deficit in MS (17–19), seem to correlate with the subjective perceived MS-related fatigue (14, 15).

Alertness—as the central nervous activation or the intensity dimension of attention—may serve as a potential interface between sleep on the one hand and more complex psychological functions such as fatigue on the other hand. Alertness is defined as achieving and maintaining a state of high sensitivity to incoming stimuli and represents a precondition for more complex cognitive functions (20–22) and is comprised of different subprocesses (23, 24).

The general wakefulness/arousal or tonic alertness—can be measured by pupillary hippus [pupillary unrest index (PUI)] in darkness as an index of sleepiness (25) [or asthenia in terms of Ref. (5)]. Tonic alertness is designated to a state of general wakefulness without doing any task. However, three different studies found no difference in pupillomotor instability between MS patients and healthy control (HC) (26–28), but moderate negative correlations with self-reported fatigue in MS patients (27, 28).

Different studies investigating a second sub-process—intrinsic alertness—by measuring the maintenance of an optimal level of arousal for a rather short time interval by expecting a stimulus in a specific task [or fatigability in terms of Ref. (5)] (20). By using a simple reaction time task, they replicate longer reaction times in MS patients compared to HC as an indicator for reduced intrinsic alertness (29–32). A reduced intrinsic alertness was especially pronounced in high fatigued MS patients compared to low fatigued MS patients (33). The intrinsic alertness in MS deteriorates over time (31) as well as after cognitive (and physical) load (30, 34).

Performance-based measures are often confounded by quality of sleep. In MS, there is a high prevalence of moderate to severe sleep problems (up to 52%) (35, 36) and a close conceptual proximity of fatigue and sleep has recently been discussed (36–38). Up to now the relation is not well understood, but quality of sleep and fatigue may act as relevant confounders to each other (37). There is some evidence that MS patients with relevant sleep disorders scored higher in fatigue scales compared to MS patients without relevant sleep disorders (36). In addition, sleep disorders are found to be the strongest predictor for MS fatigue (36), but the correlations between them are moderate (39, 40).

The aim of the study was twofold: first, to systematically measure the three sub-processes of alertness (tonic, intrinsic and phasic alertness) in MS patients and HCs as a performance-based measure of fatigue and sleep. Second, to investigate the relationship between these performance-based measures and self-report measures of fatigue and sleep in MS patients in comparison to HC.

## Materials and Methods

### Subjects

Fifty-nine outpatients (one was excluded because of technical problems with electronical questionaires) with clinically defined MS according to the McDonald criteria (41) were recruited at clinical visits at the local MS center and by local postings. Enclosed subtypes of MS were: relapsing-remitting MS (RRMS; *n* = 55) and secondary-progressive MS (SPMS; *n* = 4). MS patients were screened for anemia or thyroid dysfunction, relevant comedication (antidepressants, tretrahydrocannabinol, or modafinil) and depressive symptoms. A group of 33 MS patients without comorbidity (MS−) of anemia, thyroid dysfunction, depressive symptomsor antidepressants were analyzed in comparison to a group of 26 MS patients with at least one of the mentioned comorbid disorder (MS+). Four patients were diagnosed with anemia (hypochromic microcytic or normochromic normocytic), two patients with a thyroid dysfunction (thyroid stimulation hormone <0.27 or >4.20), and 12 were taking medicines (antidepressants, tretrahydrocannabinol, or modafinil; one of them had an anemia, too). Inclusion criteria for both MS groups comprised no relapses or steroid treatment within in the past 2 months. Exclusion criteria was a known treated comorbid disorder (six patients with anemia, thyroid dysfunction, or depression), pregnancy (one patient), and shift work (two patients). Twenty patients decline to participate the study. The MS groups did not differ between disease modifying drugs [χ^2^(4) = 2.20, *p* = 0.821; betaferon, copaxone, tysabri, fingolimod, and others].

Forty-three age- and sex-matched HC were recruited through local postings. They were also screened for selected comorbid disorders (anemia, hypothyroidism, depressive symptoms, relevant medication). Seven HC were excluded because of anemia (3 probands), thyroid dysfunction (1 proband), anemia and thyroid dysfunction (1 proband), or depressive symptoms (2 probands) measured with the Centre for Epidemiological Studies Depression Scale (CES-D) (42) and 16 HC decline to participate. Written informed consent was obtained from each participant prior to study entry. The protocol was reviewed and approved by the local ethics committee (TU-Dresden, Faculty of Medicine), and all participants gave their informed consent prior to their inclusion in the study. Main demographic and clinical data are compiled in Table 1.

**Table 1:** Sample characteristics of MS patients (MS−) without and with comorbid (MS+) disorders in comparison to healthy controls (HC).

	MS− (*n* = 33)	MS+ (*n* = 26)	HC (*n* = 43)	*F*/χ^2^/*t*	$\eta_{p}^{2}/d$
Age	40 (11)	41 (13)	39 (12)	<1^.744^	–
Gender (female/male)	23/10	21/5	33/10	1.03^.598^	–
Education (<10/10/>10 years)	2/11/20	3/13/10	0/11/32	10.81^0.029^	–
MS course (RRMS/SPMS)	31/2	24/2	–	<1^.805^	–
Subjected sleep problems	21	19	5	32.36^.000^	–
Years since onset	10 (9)	10 (7)	–	<1^0.897^	–
Years since diagnosis	8 (6)	8 (6)	–	<1^0.962^	–
Years since fatigue	6 (6)	5 (5)	–	<1^0.682^	–
EDSS	2.8 (1.5)	3.7 (1.6)	–	4.85^0.032^	0.08
MSFC total^a^^,^^b^^,^^c^	0.4 (0.5)	−0.1 (0.6)	0.6 (0.3)	15.90^.000^	0.27

*Scale *p* value represents statistical differences between MS patients with (MS+) and without (MS−) comorbidity and healthy controls (HC)*.

*^a^In the case of unequal variances, the Welch statistic is used*.

*^b^MS+ < MS− (exact p-values are presented in the text)*.

*^c^MS+ < HC (exact *p*-values are presented in the text)*.

*Mean (SD in parentheses) or frequencies; $\eta_{p}^{2}$, eta square; *d*, Cohen’s *d*; EDSS, Expanded Disability Status Scale; RRMS, relapsing-remitting MS; SPMS, secondary-progressive MS; MSFC, Multiple Sclerosis Functional Composite, Total score of MFSC are described as *z*-standardized scores; EDSS is described as metric raw scores*.

### Assessment

#### Self-Report Measure

All participants were asked to rate fatigue by a standardized questionnaire [Modified Fatigue Impact Scale (MFIS) (43)] and by a visual analogue rating scale [on a scale from 0 to 10 how strong is fatigue at the moment; before and after the pupillographic sleepiness test (PST)]. An MFIS total score of ≥38 indicates MS-related fatigue (2).

Additionally, MS patients and HCs completed standardized questionnaires in order to control for different influences of depressive symptoms (29) [CES-D (42)], sleep [Pittsburgh quality of sleep index (PSQI) (44)], and daytime sleepiness [Epworth Sleepiness Scale (ESS) (45)] on the different fatigue measures. A CES-D total score of >23, an ESS total score of >10, and a PSQI total score of >5 indicates clinically relevant depression or daytime sleepiness and distinguishes between good and poor sleepers, respectively.

#### Performance-Based Measure

To objectify cognitive aspects of MS-related fatigue or sleep, the alertness subtest of the computerized Test of Attentional Performance [TAP (23)] was used. Alertness is designated as general wakefulness or arousal that enables a person to respond effectively to any given demand. It is essential and the basis of every attentional or cognitive performance. The test comprised a simple reaction time task lasting approximately 4.5 min. Information processing speed or mean reaction time for intrinsic (without warning signal) and phasic (with warning signal) alertness were the dependent measure. There were no significant differences between the two blocks of intrinsic and the two blocks of phasic alertness (*F*s < 1.01, *p*s > 0.368 for effects of block or interactions of block and group), and the results of the two blocks were therefore collapsed, respectively.

The PST reflected the second performance-based measure. It was performed for 11 min in a quiet and dark room between 2:00 p.m. and 7:30 p.m. The pupil diameter was measured in order to quantify the typical pupillomotor hippus as a measure for tonic alertness or for central autonomic nervous activation (46). The PUI (in mm/min) was the dependent measure. The higher the PUI, the more pronounced the pupillomotor hippus and daytime sleepiness.

For MS patients, neurological disability was rated by Kurtzke’s Expanded Disability Status Scale (EDSS) using the neurostatus tool only by EDSS-certified Neurologists (47).

### Statistical Analysis

Statistical analyses were performed with the Statistical Package for Social Sciences (SPSS^®^/IBM^®^ Version 23.0 for Windows). Cross-sectional analysis was conducted using the Chi-square test (χ^2^) and analysis of variance or *t*-tests for independent samples (ANOVA; *post hoc* test: Tukey’s honestly significant difference) with respect to demographical, neurological, psychometric, and psychological variables. In the case of unequal variances between the groups, the Welch statistic is used. The Spearman correlation coefficient was calculated to measure the strength of correlations. Significance was accepted at a level of *p* ≤ 0.05.

## Results

### Sample Characteristics

MS+ patients differed from MS− patients with regard to clinical parameters although years since diagnosis and years with fatigue were comparable (see Table 1). MS+ patients presented smaller MSFC total scores (*p* < 0.01) and higher EDSS scores compared to MS− patients. MS− patients had almost similar MSFC scores compared to HC (*p* = 0.056) whereas MS + patients presented lower scores compared to HC (*p* < 0.001).

### Self-Report Measures

An MFIS total score ≥38 was measured in 28 of 59 MS patients and by one of 43 HC (χ^2^ = 24.90, *p* < 0.001). MS patients presented higher daytime sleepiness and reduced quality of sleep compared to HC. Thirty-one of all interviewed MS patients and 14 HC had an ESS total score of >10 (χ^2^ = 4.03, *p* < 0.05), whereas 42 MS patients and 12 HC had a PSQI total score of >5 (χ^2^ = 18.70, *p* < 0.001).

Sixty-five percent of MS+ patients had a MFIS total score ≥38 in comparison to 33% of MS− patients (χ^2^ = 5.99, *p* < 0.014). The majority of MS patients (MS+ and MS−) mentioned a reduced quality of sleep with a PSQI total score >5 (61 and 85% of the MS− and MS+ patients, respectively; χ^2^ = 4.09, *p* < 0.043) and half of the MS patients in both groups had higher daytime sleepiness with an ESS total score of >10 (55 and 50% of the MS− and MS+ patients, respectively; *p* > 0.728). Only 6–8% of all MS patients met criteria for clinical fatigue with a MFIS total score ≥38 without having sleep problems (PSQI total score <5).

We found significant group effects in all self-report measures except in the daytime sleepiness (*p* = 0.139; see Table 2 for details). *Post hoc* analysis revealed higher mean scores in all fatigue rating scales and in the PSQI in MS− and MS+ patients compared to HC, respectively (*p*s < 0.01).

**Table 2:** Mean (SD) of self-report and performance-based measures for MS patients (MS−) without and with comorbid (MS+) disorders in comparison to healthy controls (HC).

	MS− (*n* = 33)	MS+ (*n* = 26)	HC (*n* = 43)	*F*	*p*	$\eta_{p}^{2}$
Fatigue rating before PST^a^^,^^b^	4.2 (2.3)	4.8 (2.4)	2.4 (2.0)	11.09	0.000	0.18
Fatigue rating after PST^a^^,^^b^	5.5 (2.4)	5.5 (2.9)	3.2 (2.2)	10.56	0.000	0.18
MFIS total^a^^,^^b^^,^^c^^,^^d^	29.1 (16.6)	41.4 (17.0)	15.1 (11.1)	28.11	0.000	0.36
MFIS physical^a^^,^^b^^,^^d^	13.6 (7.6)	19.7 (7.6)	5.9 (5.8)	33.53	0.000	0.40
MFIS cognitive^a^^,^^b^^,^^c^^,^^d^	13.3 (8.5)	18.4 (9.2)	7.7 (5.3)	16.92	0.000	0.25
PSQI total^a^^,^^b^^,^^c^^,^^d^	6.0 (3.4)	8.7 (3.8)	3.6 (2.1)	22.33	0.000	0.32
ESS total	9.6 (4.2)	9.4 (5.2)	7.7 (4.4)	2.02	0.139	0.04
CES-D^a^^,^^c^^,^^d^	10.8 (7.1)	22.6 (9.8)	9.0 (5.0)	21.21	0.000	0.39
TAP intrinsic alertness^a^^,^^b^	339 (147)	362 (94)	271 (43)	7.86	0.001	0.14
TAP phasic alertness^a^^,^^b^	333 (127)	356 (100)	268 (47)	8.54	0.000	0.15
PUI^b^	7.3 (4.1)	6.4 (3.1)	5.2 (2.4)	3.97	0.022	0.07
Pupil diameter^e^	6.1 (1.0)	5.6 (1.0)	6.4 (1.2)	4.11	0.019	0.08

*Scale *p* value represents statistical differences between MS patients without (MS−) and with (MS+) comorbidities and healthy controls (HCs)*.

*^a^MS+ > HC*.

*^b^MS−> HC*.

*^c^In the case of unequal variances the Welch statistic is used*.

*^d^MS+ > MS−, MS+ < HC (exact *p*-values are presented in the text)*.

*^e^MS+ < HC (exact *p*-values are presented in the text)*.

*$\eta_{p}^{2}$, eta square; PST, pupillographic sleepiness test; MFIS, Modified Fatigue Impact Scale; PSQI, Pittsburgh sleep quality index; ESS, Epworth Sleepiness Scale; CES-D, Centre for Epidemiological Studies Depression Scale; TAP, Test of Attentional Performance; PUI, pupillary unrest index*.

Besides the fatigue rating before and after PST (*p*s > 0.563), MS+ patients had higher scores in comparison to MS− patients. The MFIS total (*p* < 0.01) and subscores (*p*s < 0.05) as well as the PSQI (*p* < 0.01) were higher in the subgroup of MS+ patients compared to the subgroup of MS− patients.

### Performance-Based Measures

More MS patients (*n* = 27 of all 59) than HC (*n* = 8) showed abnormal PST results compared to the normative data (χ^2^ = 8.16, *p* < 0.05). The same pattern was found for the TAP. In the alertness subtest, significantly more MS patients (intrinsic alertness: 39; phasic alertness: 39) than HC (intrinsic alertness: 9; phasic alertness: 16) were below average compared to the normative sample of the TAP (intrinsic alertness: χ^2^ = 22.03, *p* < 0.001; phasic alertness: χ^2^ = 8.36, *p* < 0.01). MS− and MS + patients did not differ with respect to the frequency distribution of average or below average scores (*p*s > 0.104).

We found a significant group effect in all performance-based measures (see Table 2 for details). *Post hoc* analysis revealed a significant higher PUI in MS− patients compared to HC (*p* < 0.05) whereas the pupil diameter did not differ between these groups (*p* = 0.482). In contrast, MS + patients presented a smaller pupil diameter (*p* < 0.05) but a comparable PUI (*p* = 0.292) in comparison to HC. The MS groups did not differ from each other with respect to tonic alertness scores (*p*s > 0.212).

The ANOVA of the alertness subtest of the TAP with the two factors alertness (intrinsic vs. phasic) and group (MS−, MS + and HC) revealed a significant effect of group [*F*(2, 99) = 8.41, *p* < 0.001, $\eta_{p}^{2}=0.15$]. Subsequent analyses revealed that reaction times of MS− and MS+ patients were longer compared to HC, respectively (*p*s < 0.01). The MS groups did not differ from each other (*p*s > 0.628). There was no difference between intrinsic and phasic alertness and no interaction of alertness and group (*p*s > 0.169).

### Correlation of Self-Reports and Performance-Based Measures

The PUI correlated positively with PSQI total score in MS+ patients (*R*^2^ = 0.156, see Table 3 for more details), but not in HC or in MS− patients (*p*s > 0.080). However, the PUI correlated with the ESS total score in HC (*R*^2^ = 0.147), but not in MS patients (*p*s > 0.244).

**Table 3:** Correlations of self-report and performance-based measures for MS patients without (MS−) and with (MS+) comorbid disorders in comparison to healthy controls (HC).

	Tonic alertness			Intrinsic alertness			Phasic alertness		
	MS−	MS+	HC	MS−	MS+	HC	MS−	MS+	HC
Fatigue rating before PST	−0.032	−0.094	0.028	0.524^.001^	0.012	−0.080	0.540^.001^	−0.013	−0.098
Fatigue rating after PST	0.226	−0.170	0.077	0.496^.002^	0.297.075	−0.194	0.541^.001^	0.233	−0.158
MFIS total	0.198	−0.209	0.083	0.308^.041^	0.117	−0.123	0.296^.047^	0.202	−0.084
MFIS cognitive	0.095	−0.125	0.081	0.331^.030^	0.196	−0.188	0.325^.033^	0.254	−0.258^.047^
MFIS physical	0.214	−0.305^.065^	0.103	0.289^.052^	0.063	−0.040	0.291^.050^	0.144	0.083
PSQI total	−0.202	0.395^.023^	0.218^.080^	−0.048	−0.211	−0.004	−0.017	−0.226	−0.070
ESS	0.079	−0.142	0.383^.006^	0.037	0.314^.059^	−0.165	−0.058	0.288^.077^	−0.053

*PST, pupillographic sleepiness test; MFIS, Modified Fatigue Impact Scale; PSQI, Pittsburgh sleep quality index; ESS, Epworth Sleepiness Scale. Scale p value represents statistical significance: all ps < .10 are presented*.

The alertness subtests of the TAP correlated moderately with the fatigue rating before (*R*^2^ = 0.275 for intrinsic and *R*^2^ = 0.292 for phasic alertness) and after PST (*R*^2^ = 0.246 for intrinsic and *R*^2^ = 0.293 for phasic alertness) in MS− patients, but not in MS+ patients (*p*s > 0.075) or in HC (*p*s > 0.109). The same pattern was found for the correlation of alertness with the cognition subscale of the MFIS (*R*^2^ = 0.110 for intrinsic and *R*^2^ = 0.106 for phasic alertness) and with the MFIS total score in MS− patients (*R*^2^ = 0.095 for intrinsic and *R*^2^ = 0.088 for phasic alertness). In HC, a negative correlation of phasic alertness and the cognition subscale of the MFIS were found (*R*^2^ = 0.067). Neither a correlation with the physical subscale of the MFIS (*p*s > 0.050) nor any other correlation was significant.

## Discussion

We investigated the relationship between MS fatigue and sleep problems by systematically measuring tonic, intrinsic, and phasic alertness in MS patients and HC as a potential performance-based measure of fatigue and sleep.

Alertness—as the central nervous activation or the intensity dimension of attention—may serve as a potential interface between sleep on the one hand and more complex psychological functions such as fatigue on the other hand. We systematically analyzed the three subprocesses of alertness in our study.

First, tonic alertness or the central autonomic nervous activation was measured by the PUI. MS− patients (controlled for anemia, thyroid dysfunction, and medication) presented a higher PUI compared to HC. This is in contrast to previous studies, which did not found any differences between MS patients and HC (26–28). In contrast to previous studies, we did control for anemia, thyroid dysfunction as well as relapse at the time of testing. In accordance with earlier studies (26–28), we found no difference between MS patients with comorbidity and HC. Our data provide first evidence that comorbidities such as anemia, thyroid dysfunction, or depressive symptoms have significant influence on measures of tonic alertness. In order to further elucidate the exact nature of this interaction, comorbidities should be taken into account in future studies and clinical testing of tonic alertness.

Second, intrinsic and phasic alertness, a measure of general response readiness and the ability to increase response readiness for a short period was measured by simple reaction time tasks. Although the MS groups did not differ from each other, all MS patients (without and with comorbidity) showed a reduced intrinsic and phasic alertness compared to HC. Our data are in accordance with earlier findings and support the conclusion of reduced information processing speed as a cognitive key deficit in MS (29–32).

The second goal of this study was to investigate the relationship of performance-based measures and self-report measures of MS-related fatigue and quality of sleep in MS patients and HC. Although MS− patients had poorer performance in tonic alertness than HC, we did not detect a significant correlation with fatigue or with sleep measures in this subgroup. This is not in accordance with two earlier studies, which found moderate negative correlations with self-reported fatigue and tonic alertness in MS patients. However, both studies did not control for anemia or thyroid dysfunction or for depressive symptoms/antidepressant medication (27, 28). So it is unclear whether fatigue in these studies is potentially confounded with other fatigue causing conditions. In our study, MS+ patients showed higher self-reported fatigue and more sleep problems compared to MS− patients, and a correlation between tonic alertness and quality of sleep (measured by the PSQI) was observed. This is a new finding because no known study investigated sleep-associated problems besides daytime sleepiness. In contrast to a previous study, we found no correlation with daytime sleepiness (26).

Furthermore, intrinsic and phasic alertness (measured by the TAP) seems to be moderately associated with fatigue in MS− patients. This finding is in accordance with earlier studies, which found moderate correlations (29, 30, 32–34, 48).

However, the correlations are only moderate and the explained variance is small. That’s why future studies should investigate further confounding variables such as neurological disability (EDSS score differed between MS− and MS+ patients in our study), cognitive dysfunction, medication (of neuropathic pain or of other disease/symptoms modifying drugs) or MS-related symptoms (spasticity, urinary problems, neuropathic pain) as a potential cause of secondary fatigue. We focused on alertness as a potential psychological explanation of fatigue. Future investigations could concentrate more on the different entities of fatigue [e.g., asthenia, fatigability, worsening of symptoms cf. (5)] in order to clarify the moderate correlations with MS-related fatigue measured with conventional questionnaires and to clarify a potential relationship of the different entities between alertness on the one side and fatigue on the other side.

## Conclusion

In sum, we found significant differences between MS patients compared to HC in self-report measures (MS-related fatigue, quality of sleep) and performance-based measures (tonic, intrinsic, and phasic alertness). Tonic alertness measured by PST was higher in MS patients without comorbid disorders (MS−) compared to HC, whereas intrinsic and phasic alertness measured with the TAP differed in MS patients without and with comorbidity in comparison to HC. Intrinsic and phasic alertness measures correlated moderately with conventional fatigue ratings in MS patients.

Finally, our results add new insights into the understanding of fatigue and sleep problems in that way that somatic conditions such as anemia, endocrine dysfunction (e.g., hypothyroidism) should be considered more often in clinical practice in order to exclude primary organic dysfunctions as a reason for fatigue and sleep problems.

## Ethics Statement

The protocol was reviewed and approved by the local ethics committee (TU-Dresden, Faculty of Medicine) and all participants gave their informed consent in accordance with the Declaration of Helsinki prior to their inclusion in the study.

## Author Contributions

SK and MP had full access to all the data in the study and takes responsibility for the integrity of the data and the accuracy of the data analysis. Study concept and design: SK and MP. Acquisition of data, statistical analysis, interpretation of the data, and drafting of the manuscript: MP. Critical revision of the manuscript for important intellectual content and study supervision: TZ, SK, and MP.

## Conflict of Interest Statement

SK reports grants from Novartis Pharma GmbH, Germany, during the conduct of the study; personal fees from Biogen-Idec, personal fees from Teva Pharma, outside the submitted work. MP reports grants from Novartis Pharma GmbH, Germany, during the conduct of the study. TZ has received compensation for consulting services from Almirall, Biogen Idec, Bayer, Genzyme, GlaxoSmithKline, MSD, Merck Serono, Novartis, Sanofi, Teva, and Synthon and has received research support from Bayer, Biogen Idec, the Hertie Foundation, the Roland Ernst Foundation, the German Diabetes Foundation, Merck Serono, Novartis, Teva, and Sanofi Aventis.
